# Induced Periodontitis in Rats With Three Ligature Types: An Exploratory Study

**DOI:** 10.1002/cre2.946

**Published:** 2024-08-05

**Authors:** Natalia Chatzaki, Andreas Stavropoulos, Balazs Denes, José Cancela, Stavros Kiliaridis, Catherine Giannopoulou

**Affiliations:** ^1^ Division of Regenerative Dental Medicine and Periodontology University Clinics of Dental Medicine, University of Geneva Geneva Switzerland; ^2^ Periodontology Faculty of Odontology, Malmo University Malmo Sweden; ^3^ Department of Periodontology Blekinge Hospital Karlskrona Sweden; ^4^ Division of Conservative Dentistry and Periodontology University Clinic of Dentistry, Medical University of Vienna Vienna Austria; ^5^ Department of Periodontology School of Dental Medicine, University of Bern Bern Switzerland; ^6^ Department of Orthodontics and Dentofacial Orthopedics, Dental School, Medical Faculty University of Geneva Geneva Switzerland; ^7^ Department of Orthodontics and Dentofacial Orthopedics School of Dental Medicine, University of Bern Bern Switzerland

**Keywords:** experimental periodontitis, ligatures, metal wire, rats

## Abstract

**Background:**

The placement of ligatures in the cervical area of rat molars is considered as a predictable model to induce periodontitis.

**Objectives:**

The present explorative study aimed to compare the efficacy of metal wires (MWs), without or with sandblasting, versus silk ligatures (SLs) in inducing periodontal bone loss in rats.

**Materials and Methods:**

Twenty‐four Wistar rats were randomly divided into three groups of eight rats that received three different types of ligatures (MW, sandblasted wire [SMW], and SL) around their first right mandibular molar, while the contralateral tooth was left without the ligature and served as a control. Bone loss was assessed by measuring the distance from the cementoenamel junction (CEJ) to the bone crest at the distal aspect of the first molar on central mesiodistal sections generated from micro‐CT scans taken 24 and 35 days after ligature placement.

**Results:**

In the SL group, only in two rats the ligatures were retained until the end of the 24‐day period; in all other animals, the ligatures were lost at some time point. In the SMW, the ligatures were retained only for the 24‐day period. In the MW group, no ligatures were lost. Irrespective of the group or experimental period, the difference in the crestal bone level between ligated and control teeth was in most cases *z* < 0.20 mm, that is, in 19 out of 25 pairs of teeth. In a few cases, the bone crest was more apically located at the control teeth compared to the ligated ones (four cases each, during both 24‐ and 35‐day experimental periods).

**Conclusions:**

Bone loss was minimal during the experimental period, with no significant differences between the test and control teeth, or among the three types of ligatures. MWs, not even roughened, do not seem to be a better alternative to SLs for inducing bone loss in the experimental periodontitis model in the rat. This assumption, however, has to be confirmed in a larger, well‐powered study.

## 1 Introduction

Animal models have been widely used to study the pathogenesis of periodontal diseases (Hajishengallis, Lamont, and Graves 2015). Several small (mice, rats, and rabbits) and large (monkeys, dogs, mini‐pigs, sheep, and rabbits) animal platforms have been used (Struillou et al. 2010). Each species shows resemblance in several aspects to human periodontal anatomy, microbiology, and pathophysiology (Madden and Caton 1994); however, there are also several differences and no single model can represent all aspects of human periodontal disease (Graves et al. 2008). Rats have been extensively used in periodontal research as they show several advantages, for example, identical structure of the dentogingival complex with that in humans (Oz and Puleo 2011; Yamasaki et al. 1979), ease of handling, and low cost. They also offer the advantage of relatively easier clinical intraoral manipulations compared with the much smaller mice.

Several methods have been described to induce periodontal disease in rats, including local injection of various periodontal pathogens, local injection of bacterial endotoxins, and the placement of ligatures around the cervical area of molars (Duarte et al. 2010; Graves et al. 2012; Kantarci, Hasturk, and Van Dyke 2015; Oz and Puleo 2011). The oral inoculation model, where periodontopathogenic bacteria such as *Porphyromonas gingivalis* (Pg), *Aggregatibacter actinomycetemcomitans* (Aa), *Tannerella forsythia* (Tf), and *Treponema denticola* (Morgan and Wilson 2001) are administered orally, and the gingival injection model, where Pg lipopolysaccharide (Pg‐LPS) is injected into the gingiva to induce periodontitis, have been shown not to adequately mimic oral microflora dysbiosis; these models are commonly used in mice, where, due to size limitations, placement of ligatures is challenging (Graves et al. 2012). Placement of a ligature around the teeth induces plaque accumulation and establishment of a biofilm, leading to disruption of the gingival epithelium, inflammation within the gingival connective tissue, and enhanced osteoclastogenesis, resulting in bone loss around the experimental teeth, that is, periodontitis. The ligature‐induced periodontitis model in the rat is considered as a predictable model, in the sense that disease can consistently be induced within a few days (typically over a period of about 7–15 days) (Bezerra et al. 2000; de Molon et al. 2018; Gao et al. 2022; Graves et al. 2008; Kuhr et al. 2004). Different types of ligatures have been used to induce periodontitis, for example, silk, cotton, nylon, wire, and so on (Al Bayaty et al. 2023; Cavagni et al. 2016; Dai et al. 2016; de Souza et al. 2011; Duarte et al. 2010; Fernandes et al. 2007; Fujita et al. 2010; Kamburoğlu, Ereş, and Akgün 2019; Nogueira et al. 2017; Zhang et al. 2022; Zhou et al. 2018), each having advantages and disadvantages; for example, silk ligatures (SLs) are relatively easy to place, but they often fall off after a few days, while metal wires (MWs) are reportedly more difficult to insert/adapt place but tend to remain in place better (Liu et al. 2012; Zhang et al. 2022). Nevertheless, it has been recently reported that MWs—because they are smooth—may not collect adequate amounts of plaque for effective disease induction (Zhang et al. 2022; Zhou et al. 2018).

In this context, there is not much information about the relative effectiveness of SLs versus MWs in terms of the extent of induced bone loss within a certain time frame. Furthermore, it is not known whether increasing wire surface roughness (e.g., by means of sandblasting) may influence the extent of induced bone loss; it is well known that increased surface roughness results in faster colonization and faster plaque maturation (Quirynen and Bollen 1995) Thus, the aim of this explorative study was to compare the efficacy of three types of ligatures (i.e., silk, MW, sand‐blasted MW) in inducing periodontal breakdown in adult rats.

## 2 Materials and Methods

### 2.1 Animals

Twenty‐four Wistar rats, between 14 and 21 weeks of age, housed in the animal facility of the University of Geneva, were planned for this study. The average lifespan of a rat is about 3 years, with their adolescence ending by the end of the second month of ontogenesis; thus, a rat is considered an adult at 2 months of age (Andreollo et al. 2012). In general, after at least 1 week of acclimatization, animals were randomly divided into three equally sized groups (eight animals per group) according to the type of ligature: MW, sandblasted wire (SMW), or SL. During the whole experiment, animals received a soft diet (SAFE 150 P2,5, SAFE, France) that was prepared by mixing food powder with water at a ratio of 1:1. They were kept in cages of 4 rats per cage and their body weight was measured every 2 days to control their general physical condition. Finally, 26 animals were included (for an explanation, see the Results section); the study had been previously approved by the ethical animal experimentation committee of the Canton of Geneva under license number GE/74/15.

### 2.2 Experimental Periodontitis Model

Following a split‐mouth design, periodontitis was induced by ligating the first molar in one side of the mandible chosen at random, while the contralateral tooth was left without ligation and served as a control. Ligation was performed under general anesthesia (intraperitoneal injection with 90 mg/kg of ketamine and 10 mg/kg of xylazine for approximately 30 min). The first group received a MW (0.2 mm diameter, 3M Unitek, USA), the second group received a MW sandblasted with aluminum oxide 50 μm (Orthowalker, Switzerland) (SMW) (0.2 mm diameter, 3M Unitek, USA), and the third group received a SL (4‐0, Ethicon, Johnson & Johnson, USA). Four animals from each group were planned for euthanasia after 24 and 35 days by intraperitoneal administration of anesthesia of 0.9 mL of Ketasol, 0.5 mL of Rompum 2%, and 0.6 mL of NaCl 0.9%.

### 2.3 Microcomputed Tomography Scanning

For microcomputed tomography (micro‐CT), to avoid metal artifacts, the metal ligatures were carefully cut and removed without damaging the underlying tissues. The skulls were then placed so that the molar region was closest to the center of the scanning space to avoid distortion that occurs at the edges of the scanning space. All scans were registered using a Skyscan 1076 micro‐CT scanner (Bruker microCT, Kontich, Belgium), with identical parameters: 140 mA, 70 kV, 2945 ms of exposure, 1.0‐mm Al filter, 0.2 rotation step, 180° scan, and 9‐μm nominal resolution, as previously described (Denes et al. 2013). The scans were then exported in DICOM format and analyzed with Osirix (Osirix image analysis software, Geneva, Switzerland) after being reoriented according to the region of interest, that is, the interproximal space between the first and the second molar. Central mesiodistal sections, where the distal root of the first molar and the mesial root of the second molar were best visualized (i.e., more complete), were generated for both test and control teeth. On these sections, the crestal bone level, that is, the distance from the bone crest to the cementoenamel junction (CEJ) in the distal aspect of the first molar, was measured by two operators masked to the treatment group and ligated/non‐ligated tooth; the average of the two measurements was used to represent the tooth. Mean ± SD was calculated for each group.

## 3 Results

One animal from the SMW group, and allocated to the 35‐day experimental period, developed respiratory problems after ligature placement and died during the first day of the experiment; this animal was replaced. Another animal in the same group was deemed fragile and therefore an additional animal was operated on to ensure at least 4 animals per group per observation period. The general condition of the rest of the rats was good, as no weight loss was observed throughout the experiment. The wires were retained in all animals in the MW group during the entire experimental period, while in the SMW group, only in the animals allocated in the 24‐day period were the wires retained; in the SL group, only in two animals ligatures were retained until Day 24 (Animals 2 and 4), while none of the animals had the ligatures in place at Day 35.

**Figure 1:**
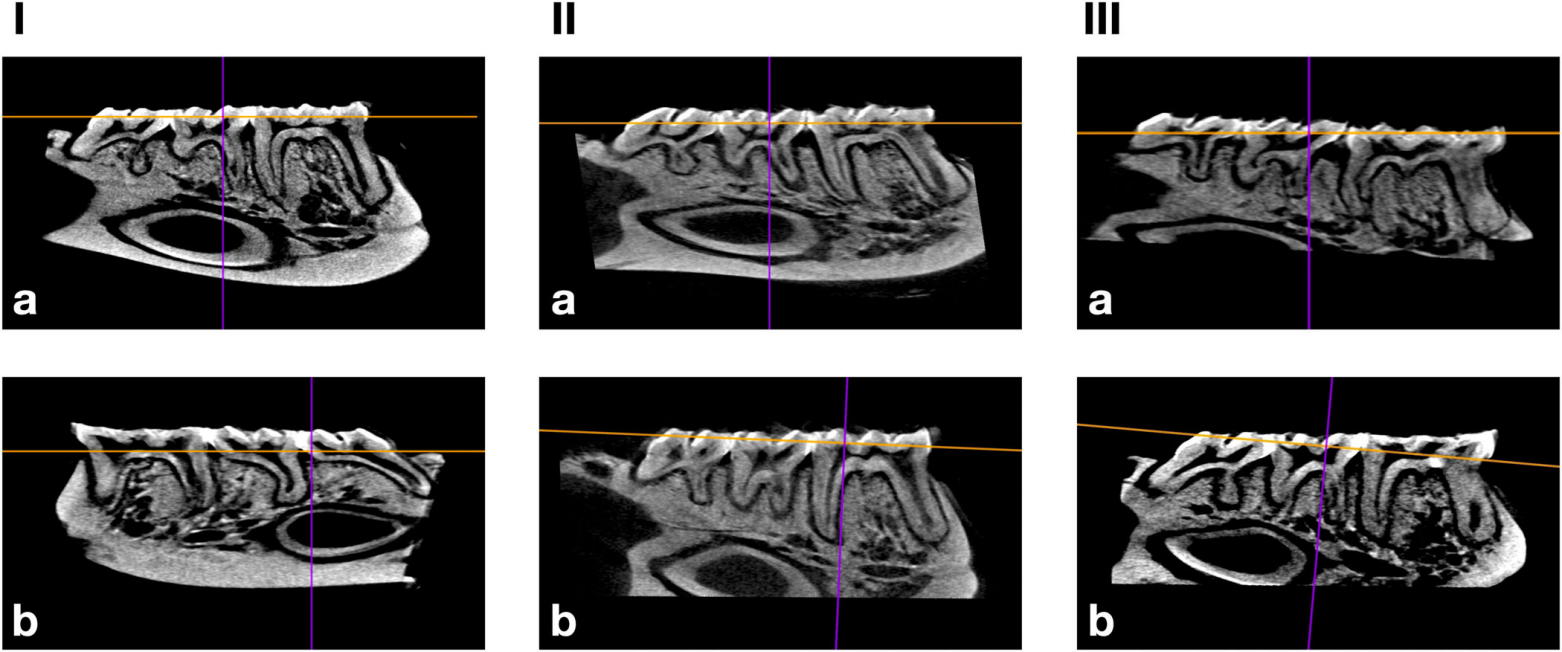
Micro‐CT scan images from the three groups: MW (I), SMW (II), and SL (III). Test sites (a) were ligated around the first molar, while control sites (b) were not ligated. In general, limited bone loss was observed irrespective of the type of ligature, and there were no remarkable differences between ligated and nonligated teeth.

The crestal bone‐level measurements in each animal in the three groups are shown in Table 1; representative images of test and control sites from the three groups are shown in Figure 1. In general, irrespective of the group or experimental period, the difference in the crestal bone level between ligated and control teeth was < 0.20 mm, that is, in 19 out of 25 pairs of teeth. Specifically, at the MW group, at 35 days, only in one animal was the bone crest in the test tooth located 0.70 mm more apically than in the control tooth; in the other three animals, the difference between test and control teeth was < 0.12 mm. Furthermore, in some cases, the bone crest was more apically located at the control teeth compared to the ligated ones during both the 24‐ and 35‐day experimental periods; however, there was no tendency for clustering of this finding in any of the groups, nor was it related to the presence or absence of the ligatures at the time of evaluation.

**Table 1:** Crestal bone levels (in mm) in the three experimental groups, in test and control sites.

	24 days			35 days		
	Test site	Control site	Difference	Test site	Control site	Difference
Group MW						
Animal 1	*0.42*	*0.55*	*−0.13* a	*0.34*	*0.23*	*0.11*
Animal 2	*0.36*	*0.35*	*0.01*	*1.03*	*0.33*	*0.70*
Animal 3	*0.45*	*0.58*	*−0.13* a	*0.43*	*0.35*	*0.08*
Animal 4	*0.83*	*0.40*	*0.43*	*0.48*	*0.36*	*0.12*
Mean (SD)	0.52 (0.21)	0.47 (0.11)		0.57 (0.31)	0.32 (0.06)	
Group SMW						
Animal 1	*0.34*	*0.24*	*0.10*	0.56	0.30	0.26
Animal 2	*0.60*	*0.26*	*0.34*	0.44	0.69	−0.25^a^
Animal 3	*0.43*	*0.40*	*0.03*	0.65	0.53	0.12
Animal 4	*0.39*	*0.53*	*−0.14* a	0.43	0.49	−0.06^a^
Animal 5	—	—		0.42	0.31	0.11
Mean (SD)	0.44 (0.11)	0.36 (0.13)		0.50 (0.10)	0.46 (0.16)	
Group SL						
Animal 1	0.47	0.31	0.16	0.49	0.37	0.12
Animal 2	*0.61*	*0.34*	*0.27*	0.34	0.43	−0.09^a^
Animal 3	0.78	0.59	0.19	0.44	0.30	0.14
Animal 4	*0.36*	*0.52*	*−0.16* a	0.46	0.51	−0.05^a^
Mean (SD)	0.55 (0.18)	0.44 (0.14)		0.43 (0.07)	0.40 (0.09)	

*Note:* Data from animals, where the ligatures were present throughout the respective experimental period, are presented in italics.

^a^ Animals where the bone crest was more apically located at the control teeth.

## 4 Discussion

The present exploratory study evaluated three different types of ligatures for inducing periodontitis in adult rats over a period of 24 and 35 days. Overall, only minute differences (commonly < 0.20 mm) in the crestal bone level were measured between test and control sites in all three groups, and there were no remarkable differences among groups. These findings were somehow surprising, as it was hypothesized that MWs, especially the sandblasted ones, which were expected to remain in place throughout the experiment and collect more plaque, would have induced more periodontal bone loss compared with the SLs, which have the tendency to fall off. Indeed, for the first 24 days of the experiment, the ligatures were retained in all animals in the MW and SMW groups, while in the SL group, only in two animals were the ligatures retained; the ligatures were retained only in the animals in the MW group for the entire duration of the experiment.

In this context, it has been recently reported that MWs, although they can be fixed more firmly and can stay longer around rat molar teeth, because of their smooth surface, seem to accumulate lower amounts of bacteria compared to threaded (e.g., silk) ligatures; this, in turn, appears to be insufficient to induce periodontal bone loss (Zhang et al. 2022). In the study by Liu et al. (2012), mentioned above, the authors described an experimental periodontitis model in rats with the use of steel ligatures, which were inoculated with Pg, to improve the efficiency of the model. In the present study, in order to increase the surface roughness of the MWs and subsequently the accumulation of plaque around the molars, we used SMWs. As already mentioned, the increase in surface roughness results in faster colonization and faster plaque maturation (Quirynen and Bollen 1995). Nevertheless, roughening of the wire ligatures did not have any measurable impact herein. In retrospect, a possible explanation may be that the wires, due to the difficulty in adapting them tightly around the very small molar teeth in the very narrow inter‐dental space, were somehow more coronally located (i.e., at a greater distance to the sulcus); thus, despite any possible impact on plaque accumulation/maturation due to roughening, a clinically measurable impact was not visible. Furthermore, roughening appears to mechanically weaken the wire, as no animal retained the ligatures in the 35‐day experimental period. The period of periodontitis reproduction for up to 35 days was quite long, also increasing the risk of ligature loss, simply due to the normal activity/function of the animal (e.g., chewing, swallowing, etc). In this context, some authors have used the second molar as an experimental site; the presence of the mesial and distal contact points at the second molar may decrease the risk of the ligature sliding (Tomina et al. 2022). However, placement of ligatures without inducing trauma to the tissues is rather complicated at the second molar, which is why the first molar was chosen in the present study.

As mentioned earlier, the challenge with the use of silk sutures—commonly reported in the literature—concerns the retention of the ligature around the teeth throughout the study. Indeed, in our study, only in two rats—of those receiving SLs—was the ligature retained for 24 days. For example, Zhang et al. (2022) reported that due to the smooth surface of the teeth and the softness of the ligature, it is technically difficult to achieve firm fixation of the ligature to the tooth, and SLs may be easily dislodged due to the powerful chewing activity of rats. A common approach for controlling the position of the ligature is anesthetizing the animals regularly. Nevertheless, this procedure is not only cumbersome but also very stressful for the animals and may give rise to ethical concerns. Yet, the increased rate of ligature loss poses a challenge in this model, since, on the one hand, the number of animals should be increased to compensate for dropouts, but on the other hand, the 3Rs rule (replacement, reduction, and refinement) (Russell and Burch 1959) should be implemented.

It the present study, relatively limited periodontal bone loss was observed in both test and control groups within the time frame chosen. In the literature, different periods of inducing experimental periodontitis have been evaluated. In a study by Duarte et al. (2010), using a cotton ligature, a period of 42 days was chosen, and bone loss was clearly evident in the teeth that had received ligatures. Kuhr et al. (2004) used SLs and evaluated bone loss after periods of 15, 30, and 60 days. It was reported that periodontal bone loss was larger over time, but the most pronounced loss occurred during the first 15 days, whereas stagnation was observed up to 60 days, indicating a diminishing effect of the ligatures. A similar pattern of periodontal bone loss has also been observed in other studies of experimentally induced periodontitis in the rat, that is, attachment and alveolar bone loss seem to occur predictably after about a 7‐day ligation period (Bezerra et al. 2000, 2002; Graves et al. 2008; Lohinai et al. 1998; Nowotny and Sanavi 1983). Thus, the time frame used in the present study was considered adequate/safe for inducing bone loss, corresponding, however, to the chronic phase of periodontal destruction. As described by de Molon et al. (2018), the process of ligature‐induced periodontitis in rats may be divided into two sequential processes: the acute phase, characterized by rapid bone destruction and the presence of inflammatory cell infiltrate, and the chronic phase, in which the number of infiltrating inflammatory cells decreases and alveolar bone resorption slows down. In this context, the negligible difference between ligated and control teeth observed in our study may also be because some periodontal bone loss had also occurred in the control sites. Indeed, it has been previously shown that depending on the type of laboratory diet and bedding conditions, variable amount of bone loss may occur (Björnsson et al. 2003) even before any experimental procedures. Furthermore, some variation in the bone levels may be due to physiological bone remodeling, after occlusal attrition, and the impact of mastication forces; it has been previously mentioned that this may be a major inherent disadvantage in all animal models of experimental periodontitis (Crawford, Taubman, and Smith 1978; Labelle and Schaffer 1966). In this context, the absence of microbiological or histological analyses in this study should be considered as a limitation of the study design; nevertheless, in an experimentally induced periodontitis study, the extent of bone loss is considered as a good surrogate of disease activity, and micro‐CT is very precise in visualizing bone loss (Stavropoulos et al. 2023).

Despite the exploratory nature of the study, and with the obvious limitations of the low number of animals per group as well as the frequent ligature loss, it is reasonable to conclude that MWs, not even roughened, do not seem to be an obvious better alternative to SLs for inducing periodontal bone loss in the rat. This assumption, however, has to be confirmed in a larger, well‐powered study.

## Author Contributions

N.C. handled data generation and data interpretation and drafted the manuscript. A.S. and C.G. were involved in data interpretation and manuscript drafting. J.C. handled data generation and reviewed the manuscript. B.D. performed experimental procedures and reviewed the manuscript. S.K. provided study ideas and handled data interpretation and manuscript reviewing.

## Ethics Statement

This study was approved by the ethical animal experimentation committee of the Canton of Geneva under license number GE/74/15.

## Conflicts of Interest

The authors declare no conflicts of interest.

## Data Availability

The data that support the findings of this study are available from the corresponding author upon reasonable request.
